# Enzyme-linked immunosorbent assays using virus-like particles containing mutations of conserved residues on envelope protein can distinguish three flavivirus infections

**DOI:** 10.1080/22221751.2020.1797540

**Published:** 2020-07-28

**Authors:** Wen-Yang Tsai, Kaitlin Driesse, Jih-Jin Tsai, Szu-Chia Hsieh, Robert Sznajder Granat, Olivia Jenkins, Gwong-Jen Chang, Wei-Kung Wang

**Affiliations:** a Department of Tropical Medicine, Medical Microbiology and Pharmacology, John A. Burns School of Medicine, University of Hawaii at Manoa, Honolulu, HI, USA; b Tropical Medicine Center, Kaohsiung Medical University Hospital, Kaohsiung, Taiwan; c Division of Infectious Diseases, Department of Internal Medicine, Kaohsiung Medical University Hospital, Kaohsiung, Taiwan; d School of Medicine, College of Medicine, Kaohsiung Medical University, Kaohsiung, Taiwan; e Faculty of Medicine and Health Sciences, Linköping University, Linköping, Sweden; f Division of Vector-Borne Diseases, Center for Disease Control and Prevention, US Department of Health and Human Service, Fort Collins, CO, USA

**Keywords:** Zika virus, flavivirus, virus-like particles, serodiagnosis, fusion loop

## Abstract

The recent outbreaks of Zika virus (ZIKV) in flavivirus-endemic regions highlight the need for sensitive and specific serological tests. Previously we and others reported key fusion loop (FL) residues and/or BC loop (BCL) residues on dengue virus (DENV) envelope protein recognized by flavivirus cross-reactive human monoclonal antibodies and polyclonal sera. To improve ZIKV serodiagnosis, we employed wild type (WT) and FL or FL/BCL mutant virus-like particles (VLP) of ZIKV, DENV1 and West Nile virus (WNV) in enzyme linked immunosorbent assays (ELISA), and tested convalescent-phase serum or plasma samples from reverse-transcription PCR-confirmed cases with different ZIKV, DENV and WNV infections. For IgG ELISA, ZIKV WT-VLP had a sensitivity of 100% and specificity of 52.9%, which was improved to 83.3% by FL/BCL mutant VLP and 92.2% by the ratio of relative optical density of mutant to WT VLP. Similarly, DENV1 and WNV WT-VLP had a sensitivity/specificity of 100%/70.0% and 100%/56.3%, respectively; the specificity was improved to 93.3% and 83.0% by FL mutant VLP. For IgM ELISA, ZIKV, DENV1 and WNV WT-VLP had a specificity of 96.4%, 92.3% and 91.4%, respectively, for primary infection; the specificity was improved to 93.7–99.3% by FL or FL/BCL mutant VLP. An algorithm based on a combination of mutant and WT-VLP IgG ELISA is proposed to discriminate primary ZIKV, DENV and WNV infections as well as secondary DENV and ZIKV infection with previous DENV infections; this could be a powerful tool to better understand the seroprevalence and pathogenesis of ZIKV in regions where multiple flaviviruses co-circulate.

## Introduction

Although the transmission of Zika virus (ZIKV) in the Americas has greatly declined since late 2017, the concerns of congenital Zika syndrome (CZS) and its potential re-emergence in flavivirus-endemic regions underscore the need for sensitive and specific diagnostic tests [1,2]. Based on the guidelines from the US Centers for Disease Control and Prevention, a positive nucleic acid test as soon as possible post-symptom onset (PSO) can confirm ZIKV infection, and a negative IgM test can exclude ZIKV infection [3]. Most (∼80%) ZIKV infections are asymptomatic; ZIKV can be transmitted following asymptomatic infection or through sexual contact [1,2]. Moreover, many individuals test for ZIKV infection beyond the period when RNA is detectable, thus serological tests remain as a critical component of ZIKV diagnosis [1–3].

In the genus *Flavivirus* of the family *Flaviviridae*, several mosquito-borne viruses belonging to different serocomplexes cause significant human diseases, including the four serotypes of dengue virus (DENV) in the DENV serocomplex, West Nile virus (WNV) and Japanese encephalitis virus (JEV) in the JEV serocomplex, yellow fever virus (YFV) as a single member, and ZIKV [4]. The envelope (E) protein, present on the surface of virions, is the major target of antibody response following flavivirus infection [4]. Traditional serological tests have been developed using recombinant E protein, inactivated virions or virus-like particles (VLP) [4–6]. However, these E protein-based serological tests are hampered by cross-reactivity among different flaviviruses. Thus, positive or equivocal results of IgM tests require further testing with time-consuming plaque reduction neutralization tests (PRNT) [3]. PRNT can confirm ZIKV infection for those who acquire ZIKV as the first flavivirus infection, so-called primary ZIKV (pZIKV) infection, but can only be interpreted as unspecified flavivirus infections for those who have had other flavivirus infections in the past, restricting its application for ZIKV serodiagnosis in flavivirus-endemic regions.

The ectodomain of E protein contains three domains (I, II and III). The fusion loop (FL) is located at the tip of domain II and contains several residues absolutely conserved among different flaviviruses; the BC loop (BCL), next to FL, also contains several highly conserved residues [4,7]. Previously, we and others have shown that a significant proportion of anti-E antibody in serum are cross-reactive and recognize FL residues [8–10]. Moreover, our study of 32 flavivirus-cross reactive human monoclonal antibodies identified several key FL residues (such as W101 and F108) and/or BCL residues (such as T76, Q77, G78 and E79) as epitopes [11], suggesting that FL and/or BCL residues contribute to the cross-reactivity of E protein-based serological tests [11–15].

Co-expression of flavivirus premembrane (prM) and E proteins can generate VLP, which are similar to the infectious virions in the biophysical and antigenic features. Several studies have employed flavivirus VLP to study the function of prM/E proteins, particle assembly, serodiagnostic antigens and vaccine candidates [16–21]. Moreover, several potently neutralizing human monoclonal antibodies against flaviviruses have been shown to recognize only conformational and quaternary epitopes present on virion or VLP but not on recombinant E protein [22–25], making VLP an ideal and non-infectious antigens for serological tests compared with recombinant E protein.

Different approaches have been employed to overcome such cross-reactivity. One is to use nonstructural protein 1 (NS1) as antigen. Several ZIKV NS1 protein-based enzyme-linked immunosorbent assays (ELISA) or microsphere immunoassay (MIA) including a recently reported blockade of binding ELISA have shown improved specificity [26–32]. However, the durability of anti-NS1 antibodies in serum remains a challenge for seroprevalence study. The second approach is to use recombinant domain III (DIII) as antigen; one study reported reduced cross-reactivity by recombinant DIII of ZIKV and DENV2, but not for secondary DENV (sDENV) infection during early convalescence [33]. Another approach is to use FL- and/or BCL-mutated recombinant E protein or VLP as antigen. Previous studies using such approach have reported reduced cross-reactivity for DENV, WNV, JEV and ZIKV in ELISA [20,21,34–36], nonetheless, the assays developed from these studies primarily targeted one or two flaviviruses. Another study using FL/BCL-mutated recombinant E proteins of ZIKV and DENV1-4 showed improved specificity in IgM ELISA, however, cross reactivity in IgG ELISA remains, which requires pre-incubation with large amounts of heterologous recombinant E protein to reduce [37].

After the ZIKV outbreak in the Americas, several questions remain unanswered such as its surveillance, seroprevalence and interactions with other flaviviruses in endemic regions [2,3]. Recent studies have shown that DENV- or WNV-immune sera enhanced ZIKV replication in vitro and in mice [38–43]. However, such enhancement was not observed in non-human primates, highlighting the importance of more human studies in the field [44]. Two cohort studies reported that prior DENV infection was associated with decreased risk of symptomatic ZIKV infection [45,46]. To advance this area of research, serological tests that can discriminate different DENV and ZIKV infections including primary DENV, sDENV, pZIKV and ZIKV infection with previous DENV (ZIKVwprDENV) infections, as well as other flavivirus infections are critically needed to better understand the epidemiology and pathogenesis of ZIKV in endemic regions.

In this study, we employed wild type (WT) and FL or FL/BCL mutant VLP of ZIKV, DENV1 and WNV to develop IgG and IgM ELISA and investigated convalescent- and post-convalescent-phase serum or plasma samples from reverse transcription-PCR (RT–PCR) confirmed cases with different ZIKV, DENV and WNV infections. We found that FL or FL/BCL mutant VLP and the ratio of relative optical density (rOD) of mutant to WT VLP can improve the specificity of IgG ELISA, and proposed an algorithm to discriminate three flavivirus infections.

## Materials and methods

### Ethics statement and human samples

The study was approved by the Institutional Review Board (IRB) of the University of Hawaii (CHS #17568, CHS#23786). The numbers, serotypes, sampling time and sources of different panels of serum or plasma samples are summarized in Table S1. Samples collected <3 or ≥3 months PSO were designated as convalescent- or post-convalescent-phase samples, respectively; for pDENV1 panel < or ≥4 months was used to separate the two subgroups with comparable samples size. Samples from RT–PCR confirmed Zika cases were from the Pediatric Dengue Cohort Study and the Pediatric Dengue Hospital-based Study in Managua, Nicaragua between July 2016 and March 2017 as described previously [47,48]. These studies were approved by the IRBs of the University of California, Berkeley, and Nicaraguan Ministry of Health. Thirty-six plasma samples from blood donors, who were WNV-positive by the transcription-mediated amplification, IgM and IgG antibody tests between 2006 and 2015, were designated as pWNV infection panel and provided by the American Red Cross at Gaithersburg, Maryland [49]. Samples from RT–PCR confirmed cases with pDENV1 or sDENV infection were from Taiwan, Hawaii and Nicaragua prior to the 2015–2016 Zika outbreak; 66 flavivirus-naïve samples from a seroprevalence study were included as control [10,50–52]. pDENV1 or sDENV infection was determined by IgM/IgG ratio or focus-reduction neutralization tests as described previously [50–52].

### Generation of plasmid constructs and VLP

The plasmids expressing WT premembrane (prM)/E proteins of DENV1 (Hawaii strain) and prM/E proteins with two key FL mutations (W101A and F108A) have been described previously [10,11]. The plasmids expressing WT prM/E proteins of WNV (NY99 strain), ZIKV (PRVABC59 strain), and ZIKV (MR766 strain) were generated by cDNA synthesis using RNA extracted from culture supernatants, followed by PCR as described previously [10,11]. After digestion with KpnI and NotI, the PCR products were cloned into respective sites of pCB vector. Plasmids containing FL mutations and/or BCL mutations were generated by polymerase incomplete primer extension PCR method with two reverse primers overlapping on the mutation sites [53]. After PCR, reaction mixtures were directly transformed into DH5α competent cells for selection. Table S1 summarizes the plasmids generated in this study. All constructs were confirmed by sequencing the entire inserts to rule out second site mutations. The sequences of primers used will be provided upon request.

To generate VLP, 293 T cells (1 × 10^5^ cells) were transfected with 10 μg of plasmid DNA. At 48 h, culture supernatants were collected, clarified by centrifugation at 1250× *g* for 20 min, filtered through a 0.22 μm pore-sized membrane (Sartorius), layered over a 20% sucrose buffer, and ultracentrifuged at 65,000× *g* at 4°C for 5 h to obtain pellets containing VLP, which were resuspended in 30 μl TNE buffer [10,11].

### Igg and IgM ELISA

WT and mutant VLP were subjected to serial two-fold dilutions (1:200 to 1:6400), coated on 96-well plates and tested with positive control serum to determine the titre, which was the highest dilution to reach optical density (OD) of 1. For IgG ELISA, the titrated WT and mutant VLP (such as 1:1600) were coated onto 96-well plates at 4°C overnight, followed by blocking (StartingBlock buffer, Thermo Scientific, Waltham, MA), incubation with primary antibody (serum or plasma at 1:400) and secondary antibody (anti-human IgG conjugated with horseradish peroxidase, Jackson Immune Research Laboratory, West Grove, PA), and wash [30,31]. After adding tetramethylbenzidine substrate (Thermo Scientific, Waltham, MA) and stop solution, the OD at 450 nm was read with a reference wavelength of 630 nm. Each ELISA plate included two positive controls (confirmed-ZIKV, DENV or WNV infection), four negative controls (flavivirus-naïve), and samples (all in duplicate). The OD values were divided by the mean OD value of one positive control (OD close to 1) in the same plate to calculate the rOD values for comparison between plates [30,31]. The cutoff rOD of each plate was the mean rOD value of negatives plus 12 standard deviations, which gave a confidence level of 99.9% from 4 negatives [54]; the mean cutoff rOD of all plates for the same antigen was used as the final cutoff rOD. Each ELISA was performed twice (each in duplicate). IgM ELISA was performed similarly except each sample was incubated with Gullsorb reagent (Meridian Bioscience), an IgG absorbent, for 10 min before adding to wells [30,31].

### Statistical analysis

Two-tailed Mann–Whitney test was used to determine the *P* values between two groups, and the receiver-operating characteristics (ROC) analysis the cutoffs of the rOD ratios (GraphPad Prism 6). The 95% confidence interval (CI) was calculated by Excel.

## Results

### WT and FL or FL/BCL mutant VLP

We first generated two ZIKV VLP (based on PRVABC59 and MR766 strains) and tested with pZIKV, primary WNV (pWNV) and primary DENV1 (pDENV1) infection panels in IgG ELISA. In agreement with the notion that different ZIKV strains belong to a single serotype [55], comparable detection rates and rOD values were found between ZIKV VLP derived from the two strains (Figure S1). ZIKV VLP derived from the PRVABC59 strain, designated as ZIKV WT-VLP, which was close to the circulating strain, was chosen for this study. To determine if FL alone or FL/BCL mutations can reduce the cross-reactivity, we tested ZIKV WT-VLP, FL-VLP containing key FL mutations (W101A and F108A), and FL/BCL-VLP containing FL (W101A and F108A) and 3 BCL (T76A, Q77A and G78A) mutations. Compared with WT- and FL-VLP, FL/BCL-VLP greatly reduced the cross-reactivity from the pWNV panel (6/36 positive vs 28/36 and 11/36 positive) (Figure S2(A–C)). We also generated FL/4BCL-VLP, which contained additional BCL residue mutation (E79A) but did not further reduced cross-reactivity (Figure S2(D–E)). Due to its low expression, FL/4BCL-VLP was not used. For DENV1 and WNV VLP, initial testing revealed significant reduction of cross-reactivity by FL-VLP; thus, DENV1 and WNV FL-VLP were used in this study (Table S2). We have also examined serial dilutions of each mutant-VLP in comparison with WT-VLP (ZIKV FL/BCL-VLP vs WT-VLP, DENV1 FL-VLP vs WT-VLP, and WNV FL-VLP vs WT-VLP) and their binding to different antibodies in IgG ELISA. The comparable binding of mutant- and WT-VLP at similar dilutions to different flavivirus-immune sera and mAbs except the FL mAbs suggested that each mutant-VLP was expressed well and maintained the overall antigenic structure (Figure S3). However, the quaternary structure of mutant-VLP cannot not be assessed due to the lack of using quaternary epitope mAbs.

### Reduction of cross-reactivity of ZIKV and DENV1 IgG ELISA by FL or FL/BCL VLP and the rOD ratio of mutant to WT VLP

**Figure 1.**
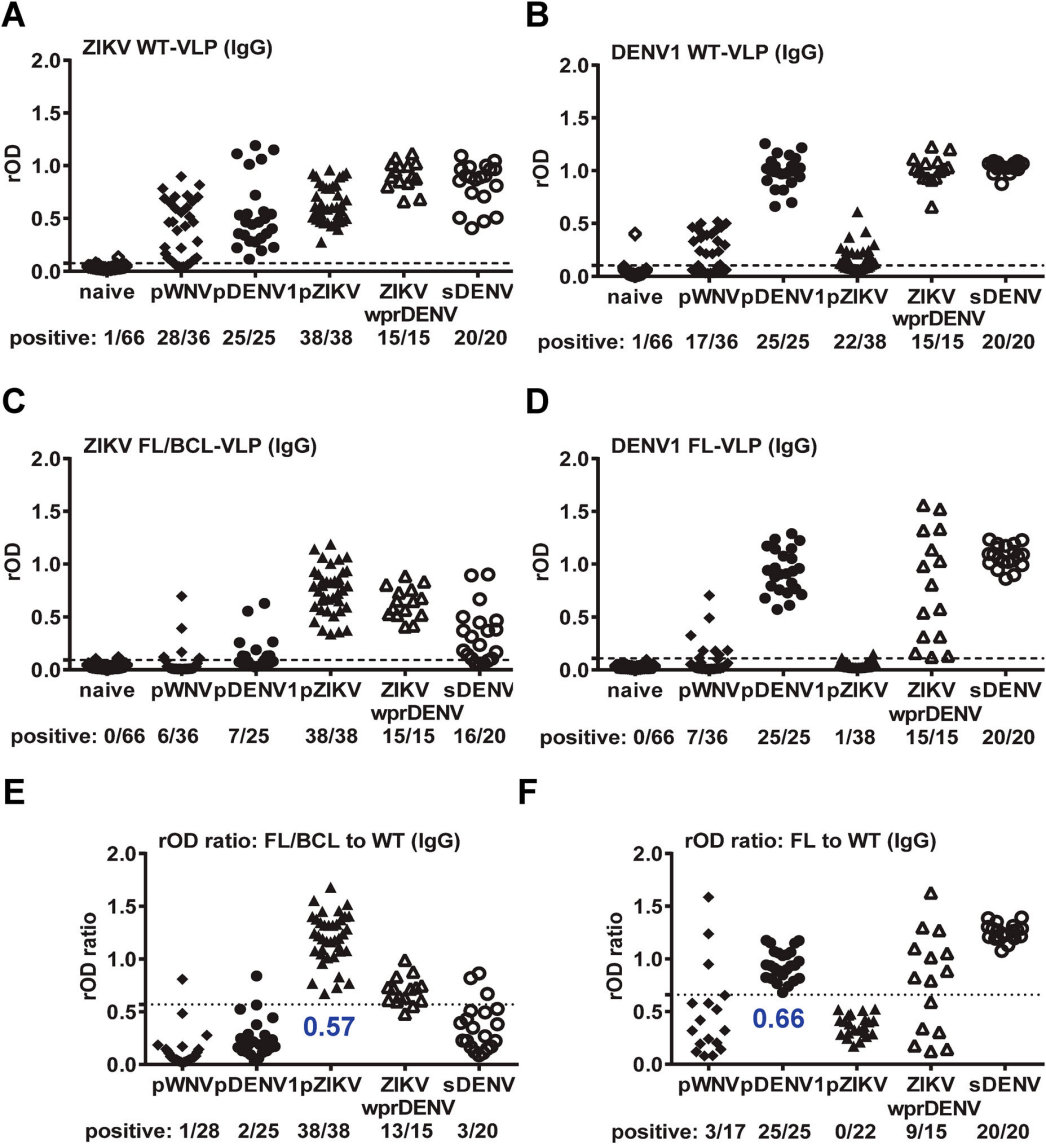
Results of ZIKV and DENV1 VLP IgG ELISA. Convalescent- and post-convalescent-phase serum or plasma samples from different panels were tested with ZIKV (A,C,E) and DENV1 (B,D,F) VLP IgG ELISA. (A,B) WT-VLP, (C,D) FL/BCL- or FL-VLP, (E,F) rOD ratio of mutant to WT VLP. Data are the means of two experiments (each in duplicate). Dashed lines indicate cutoff rOD and dotted lines cutoff rOD ratio in panels E and F.

We next tested IgG ELISA of ZIKV WT-VLP with different panels, including pWNV, pDENV1, pZIKV, ZIKVwprDENV and sDENV infections as well as flavivirus-naïve samples. Compared with flavivirus-naïve panel, ZIKV WT-VLP can be recognized by both ZIKV (pZIKV and ZIKVwprDENV) and non-ZIKV (pWNV, pDENV1 and sDENV) samples (Figure 1(A)). The cross-reactivity from pWNV and pDENV1 panels was greatly reduced by FL/BCL-VLP (Figure 1(C)). Based on the ROC analysis, we used the ratio of rOD of mutant to WT VLP and compared between ZIKV and non-ZIKV panels to determine a cutoff of 0.57, in which the cross-reactivity from non-ZIKV panels was further reduced (Figure 1(E)). Interestingly, the ZIKVwprDENV and sDENV panels can be discriminated with a sensitivity/specificity of 86.7%/85%. Similar trend was observed for DENV1 IgG ELISA in that the cross-reactivity to WT-VLP from pWNV and pZIKV panels was greatly reduced by FL-VLP and by the rOD ratio of mutant to WT VLP with a cutoff of 0.66 (Figures 1(B, D, F)). Notably, the detection rate for ZIKVwprDENV panel was decreased by the rOD ratio, suggesting reduced recognition of DENV1 FL-VLP by some ZIKVwprDENV samples.

### Reduction of cross-reactivity of ZIKV and DENV1 IgM ELISA by FL or FL/BCL VLP

**Figure 2.**
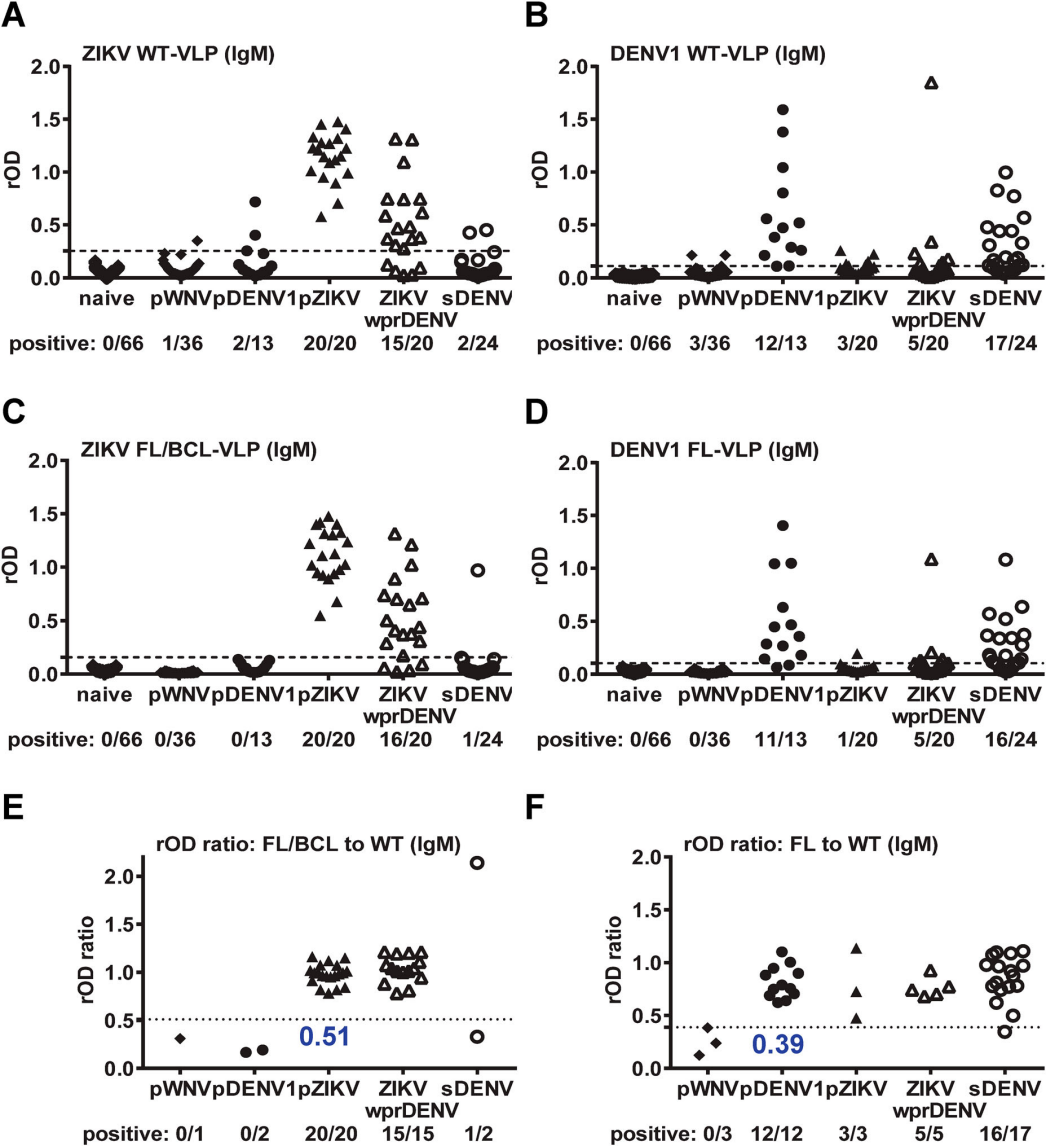
Results of ZIKV and DENV1 VLP IgM ELISA. Convalescent-phase samples from different panels were tested with ZIKV (A,C,E) and DENV1 (B,D,F) VLP IgM ELISA. (A,B) WT-VLP, (C,D) FL/BCL- or FL-VLP, (E,F) rOD ratio of mutant to WT VLP. Data are the means of two experiments (each in duplicate). Dashed lines indicate cutoff rOD and dotted lines cutoff rOD ratio in panels E and F.

We further tested IgM ELISA with different panels of convalescent-phase samples. Compared with IgG ELISA in Figure 1(A), ZIKV WT-VLP in IgM ELISA were recognized mainly by ZIKV samples (pZIKV and ZIKVwprDENV) with cross-reactivity to a few non-ZIKV samples (1 pWNV, 2 pDENV1 and 2 sDENV) (Figure 2(A)), which was slightly reduced by ZIKV FL/BCL-VLP or by the rOD ratio with a cutoff of 0.51 (Figure 2(C, E)). Similarly, compared with IgG ELISA in Figure 1(B), DENV1 WT-VLP in IgM ELISA were recognized mainly by DENV samples (pDENV1, sDENV) with some cross-reactivity to non-DENV or non-recent DENV samples (3 pWNV, 3 pZIKV and 5 ZIKVwprDENV) (Figure 2(B)); the cross-reactivity was slightly reduced by DENV1 FL-VLP but not by the rOD ratio with a cutoff of 0.39 (Figure 2(D, F)).

### Reduction of cross-reactivity of WNV IgG and IgM ELISA by FL VLP

**Figure 3.**
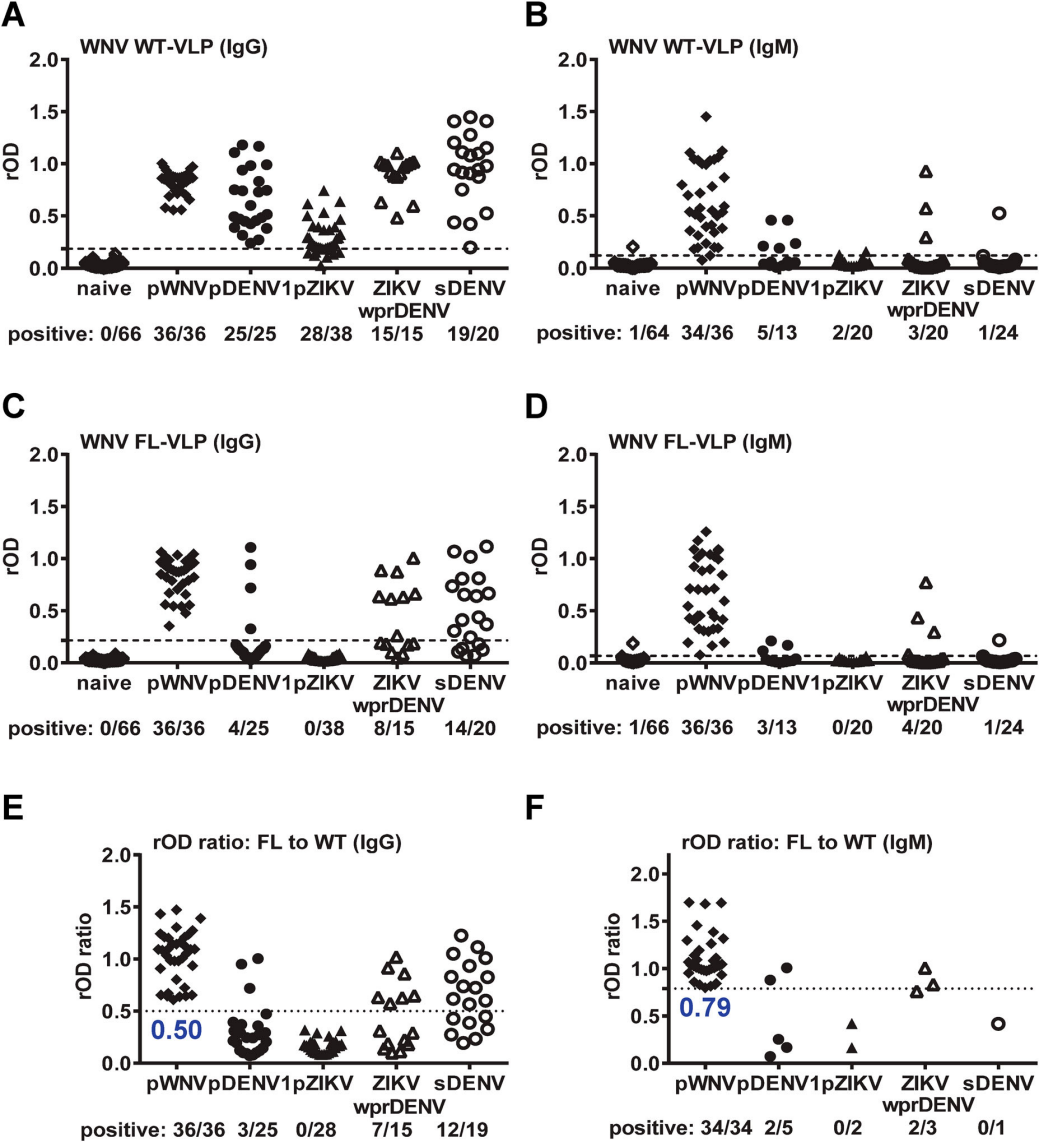
Results of WNV VLP IgG and IgM ELISA. Convalescent- and post-convalescent-phase samples from different panels were tested with WNV VLP IgG ELISA (A,C,E), and convalescent-phase samples with WNV VLP IgM ELISA (B,D,F). (A,B) WT-VLP, (C,D) FL-VLP, and (E,F) rOD ratio of mutant to WT VLP. Data are the means of two experiments (each in duplicate). Dashed lines indicate cutoff rOD and dotted lines cutoff rOD ratio in panels E and F.

We next tested IgG and IgM ELISA of another flavivirus, WNV. The cross-reactivity to WNV WT-VLP IgG ELISA from pZIKV and pDENV1 panels was reduced by WNV FL-VLP or by the rOD ratio with a cutoff of 0.50 (Figure 3(A, C, E)), however, considerable cross-reactivity from panels with repeated flavivirus infection (sDENV and ZIKVwprDENV) remains (Figure 3(C, E)). Compared with IgG ELISA, WNV WT-VLP in IgM ELISA were recognized mainly by pWNV samples with some cross-reactivity to non-WNV samples (5 pDENV1, 2 pZIKV, 3 ZIKVwprDENV and 1 sDENV) (Figure 3(B)); the cross-reactivity was slightly reduced by WNV FL-VLP or by the rOD ratio with a cutoff of 0.79 (Figure 3(D, F)).

### Sensitivity and specificity

Table 1 summarizes the sensitivity and specificity of different IgG ELISA. Comparing WT- and FL- or FL/BCL-VLP ELISA, the overall sensitivity is high (100%). The specificity of WT-VLP ELISA (52.9 to 70.0%) were lower than that of FL- or FL/BCL-VLP ELISA (83.0 to 93.3%). The specificity of ZIKV WT-VLP ELISA (52.9%) was improved to 83.3% by FL/BCL-VLP and to 92.2% by the rOD ratio of mutant to WT VLP. Similarly, the specificity of DENV1 WT-VLP ELISA (70.0%) was improved to 93.3% and 91.4% by FL-VLP and the rOD ratio, respectively. For WNV WT-VLP ELISA, the specificity (56.3%) was improved to 83.0% by FL-VLP and to a less extent to 66.1% by the rOD ratio, probably due cross-reactivity from panels with repeated flavivirus infections (sDENV and ZIKVwprDENV) as described above.

**Table 1.** Sensitivity and specificity of different VLP-IgG ELISA.

ELISA^a^		% Sensitivity (95% CI)^b^	% Specificity (95% CI)^b,c^
ZIKV WT-VLP	Overall	100 (100–100)	52.9 (44.6–57.2)
	Subgroup	pZIKV:100, ZIKVwprDENV:100	naïve:98.5, pDENV1:0, sDENV:0, pWNV:22.2
ZIKV FL/BCL-VLP	Overall	100 (100–100)	**83.3** (77.1–86.5)
	Subgroup	pZIKV:100, ZIKVwprDENV:100	naïve:100, pDENV1:93.8, sDENV:20.0, pWNV:83.3
rOD ratio ≥0.57 (ZIKV FL/BCL to WT VLP)	Overall	93.9 (85.8–98.1)	**92.2** (85.6–95.5)
	Subgroup	pZIKV:100, ZIKVwprDENV:86.7	naïve:NA, pDENV1:93.8, sDENV:85.0, pWNV:96.4
DENV1 WT-VLP	Overall	100 (100–100)	70.0 (61.8–74.2)
	Subgroup	pDENV1:100, sDENV:100, ZIKVwprDENV:100	naïve:98.5, pZIKV:0, pWNV:52.8
DENV1 FL-VLP	Overall	100 (100–100)	**93.3** (88.9–95.6)
	Subgroup	pDENV1:100, sDENV:100, ZIKVwprDENV:86.7	naïve:100, pZIKV:94.4, pWNV:80.1
rOD ratio ≥0.66 (DENV1 FL to WT VLP)	Overall	88.2 (79.4–92.8)	**91.4** (82.2–96.2)
	Subgroup	pDENV1:100, sDENV:100, ZIKVwprDENV:60.0	naïve:NA, pZIKV:100, pWNV:82.4
WNV WT-VLP	Overall	100 (100–100)	56.3 (47.9–60.6)
	Subgroup	pWNV:100	naïve:100, ZIKVwprDENV:0, pZIKV:50.0, pDENV1:0, sDENV:5.0
WNV FL-VLP	Overall	100 (100–100)	**83.0** (76.6–86.2)
	Subgroup	pWNV:100	naïve:100, ZIKVwprDENV:46.7, pZIKV:100, pDENV1:93.8, sDENV:30.0
rOD ratio ≥0.50 (WNV FL to WT VLP)	Overall	100 (100–100)	**66.1** (54.0–72.3)
	Subgroup	pWNV:100	naïve:NA, ZIKVwprDENV:53.3, pZIKV:100, pDENV1:93.8, sDENV:36.8

^a^BCL: BC loop; CI, confidence interval; ELISA: enzyme-linked immunosorbent assays; FL: fusion loop; NA: not applicable; pDENV1: primary dengue virus type 1 infection; pWNV: primary West Nile virus infection; pZIKV: primary Zika virus infection; rOD: relative optical density; sDENV: secondary dengue virus infection; Sens: sensitivity; Spec: specificity; VLP: virus-like particles; WT: wild type; ZIKVwprDENV: Zika virus infection with previous dengue virus infection.

^b^For simplicity, the 95% CIs in the subgroup are not shown.

^c^Compared to that of WT-VLP, the improved specificity by FL-VLP, FL/BCL-VLP or rOD ratio is bolded.

Table 2 summarizes the sensitivity and specificity of different IgM ELISA. The sensitivity of ZIKV WT-VLP for pZIKV panel (100%) was higher than that for ZIKVwprDENV panel (75%), suggesting higher sensitivity of IgM ELISA for primary infection. Similarly, the sensitivity of DENV1 WT-VLP for pDENV1 panel (100%) was higher than that for sDENV panel (70.8%). Comparing WT- and FL- or FL/BCL-VLP IgM ELISA, the sensitivity for primary infection panels (pZIKV, pDENV1 and pWNV) is high (91.7 to 100%). The overall specificity of IgM ELISA (91.4 to 99.3%) is higher than that of IgG ELISA (52.9 to 93.3%). Interestingly, the specificity IgM ELISA based on WT-VLP was improved by FL- or FL/BCL-VLP, from 96.4% to 99.3%, 92.3% to 95.8%, and 91.4% to 93.7% for ZIKV, DENV1 and WNV, respectively. The specificity of IgM ELISA was not improved by the rOD ratio.

**Table 2.** Sensitivity and specificity of different VLP-IgM ELISA.

ELISA^a^		% Sensitivity (95% CI)^b^	% Specificity (95% CI)^b,c^
ZIKV WT-VLP	Overall	87.5 (77.3–92.7)	96.4 (93.3–98.0)
	Subgroup	pZIKV:100, ZIKVwprDENV:75.0	naïve:100, pDENV1:83.3, sDENV:91.7, pWNV:97.2
ZIKV FL/BCL-VLP	Overall	90.0 (80.7–94.7)	**99.3** (97.9–100)
	Subgroup	pZIKV:100, ZIKVwprDENV:80.0	naïve:100, pDENV1:100, sDENV:95.8, pWNV:100
rOD ratio ≥0.51 (ZIKV FL/BCL to WT VLP)	Overall	100 (100–100)	80.0 (44.9–97.9)
	Subgroup	pZIKV:100, ZIKVwprDENV:100	naïve:NA, pDENV1:100, sDENV:50.0, pWNV:100
DENV1 WT-VLP	Overall	80.6 (67.6–87.2)	92.3 (87.9–94.5)
	Subgroup	pDENV1:100, sDENV:70.8	naïve:100, ZIKVwprDENV:75.0 pZIKV:85.0, pWNV:91.7
DENV1 FL-VLP	Overall	75.0 (60.9–82.2)	**95.8** (92.5–97.5)
	Subgroup	pDENV1:91.7, sDENV:66.7	naïve:100, ZIKVwprDENV:75.0 pZIKV:95.0, pWNV:100
rOD ratio ≥0.39 (DENV1 FL to WT VLP)	Overall	96.6 (89.9–99.9)	27.3 (1.0–40.7)
	Subgroup	pDENV1:100, sDENV:94.1	naïve:NA, ZIKVwprDENV:0 pZIKV:0, pWNV:100
WNV WT-VLP	Overall	94.4 (87.0–98.3)	91.4 (87.0–98.3)
	Subgroup	pWNV:94.4	naïve:98.4, ZIKVwprDENV:85.0 pZIKV:90.0, pDENV1:58.3, sDENV:95.8
WNV FL-VLP	Overall	100 (100–100)	**93.7** (89.7–95.7)
	Subgroup	pWNV:100	naïve:98.5, ZIKVwprDENV:80.0 pZIKV:100, pDENV1:75.0, sDENV:95.8
rOD ratio ≥0.79 (WNV FL to WT VLP)	Overall	100 (100–100)	63.6 (35.2–78.1)
	Subgroup	pWNV:100	naïve:NA, ZIKVwprDENV:33.0, pZIKV:100, pDENV1:60.0, sDENV:100

^a^BCL: BC loop; CI, confidence interval; ELISA: enzyme-linked immunosorbent assays; FL: fusion loop; NA: not applicable; pDENV1: primary dengue virus type 1 infection; pWNV: primary West Nile virus infection; pZIKV: primary Zika virus infection; rOD: relative optical density; sDENV: secondary dengue virus infection; Sens: sensitivity; Spec: specificity; VLP: virus-like particles; WT: wild type; ZIKVwprDENV: Zika virus infection with previous dengue virus infection.

^b^For simplicity, the 95% CIs in the subgroup are not shown.

^c^Compared to that of WT-VLP, the improved specificity by FL-VLP or FL/BCL-VLP is bolded.

## Discussion

In this study, we utilized WT and FL or FL/BCL mutant VLP to develop IgG and IgM ELISA to detect three flavivirus infections, and found that mutant VLP and the rOD ratio of mutant to WT VLP greatly reduced the cross-reactivity and improved the specificity of IgG ELISA. Mutant VLP further improved the specificity of IgM ELISA. These findings have tremendous implications for E protein-based serodiagnosis and serosurveillance in regions where multiple flaviviruses co-circulate.

**Figure 4.**
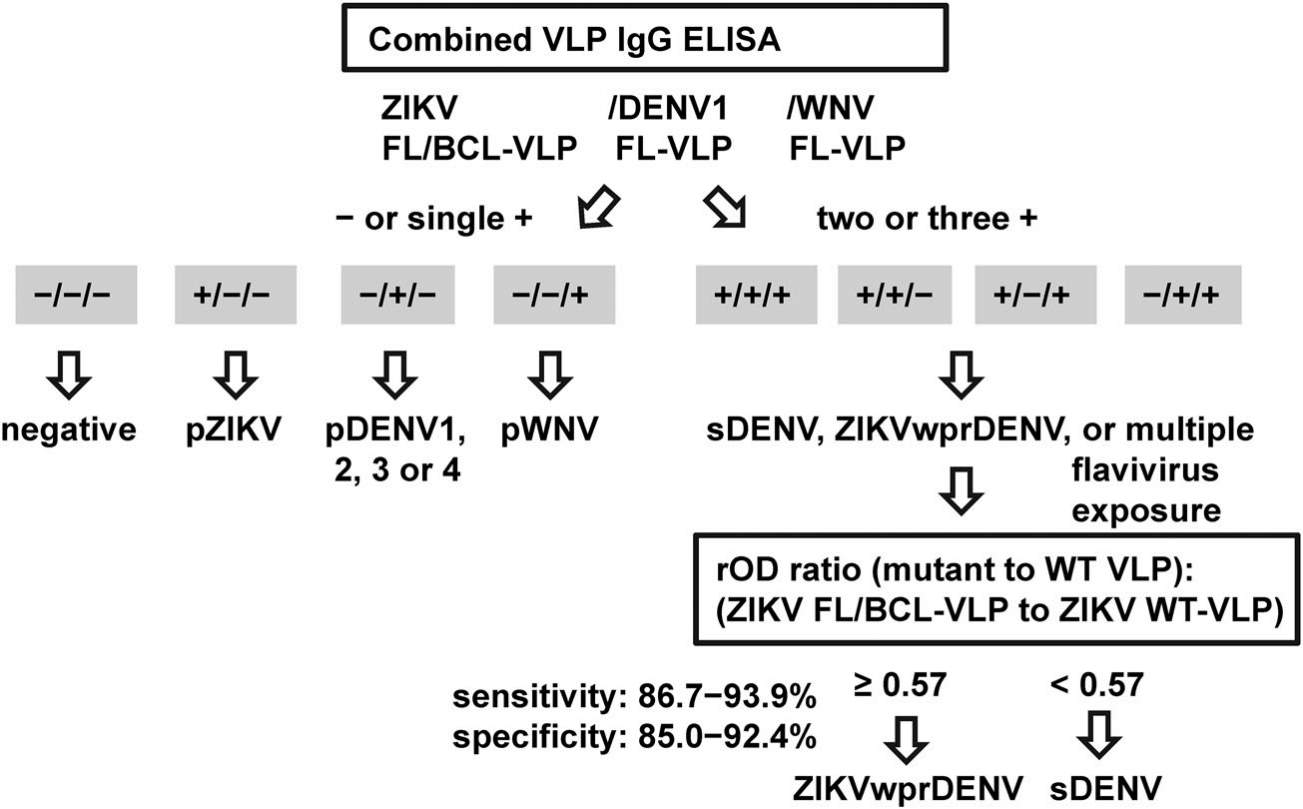
Proposed algorithm of combined VLP IgG ELISA to distinguish three flavivirus infections. Based on the positivity to FL or FL/BCL mutant VLP of three flaviviruses (ZIKV, DENV and WNV), the samples that were negative to all three or positive to one of the mutant VLP could be flavivirus naïve or primary DENV, pZIKV or pWNV infection. For samples that were positive to two or more mutant VLP, the rOD ratio of ZIKV mutant to WT VLP will be calculated to distinguish sDENV and ZIKVwprDENV infections.

For IgG ELISA, we propose to use a combination of WT and FL or FL/BCL mutant VLP to distinguish ZIKV, DENV and WNV infections. Figure 4 depicts an algorithm, in which the results of mutant VLP (ZIKV, DENV1 and WNV) will be first analyzed to identify primary DENV, pZIKV and pWNV infections, followed by the rOD ratio of ZIKV mutant to WT-VLP to discriminate ZIKVwprDENV and sDENV infections. Based on Figure 1(E), the rOD ratio of ZIKV mutant VLP to WT-VLP with a cutoff of 0.57 can distinguish ZIKVwprDENV and sDENV panels; the sensitivity/specificity was 86.7%/85% comparing these two panels and 93.9%/92.2% including other panels (Table 1). This is highly relevant to seroprevalence study in regions where both DENV and ZIKV are prevalent. Notably, the rOD ratios of DENV1 and WNV mutant to WT-VLP were not recommended due to their lower sensitivity/specificity (88.2/91.4% for DENV1 and 100/66.1% for WNV) compared with mutant VLP (Table 1).

For IgM ELISA, FL or FL/BCL mutant VLP can improve the specificity of WT VLP from 91.4−96.4% to 93.7−99.3% while maintaining a high sensitivity (91.7−100%) for primary infection panels (pZIKV, pDENV1 and pWNV) comparable to that of WT-VLP (94.4−100%) (Table 2). Thus, we propose to use FL or FL/BCL mutant VLP for IgM ELISA. In contrast to the improved specificity of IgG ELISA by the rOD ratio (Table 1), the specificity of IgM ELISA was not improved by the rOD ratio (Table 2). This is probably due to relatively few samples that cross-reacted to both WT and mutant VLP in IgM ELISA, thus lowering the specificity of the rOD ratio.

Previously, we developed combined DENV1-4 and ZIKV NS1 IgG ELISA with a sensitivity/specificity of 100/82.9% and 94.5/91.9% to detect ZIKV and DENV1 infections, respectively [31]. We have also developed a multiplex NS1 IgG MIA with a sensitivity/specificity of 100/87.9%, 87.9/99.1% and 86.1/78.4% for ZIKV, DENV1 and WNV infections, respectively [32]. Compared with these two NS1-based IgG assays, our mutant VLP IgG ELISA had a sensitivity/specificity of 100/83.3%, 100/93.3% and 100/83.0% for ZIKV, DENV1 and WNV infections, respectively (Table 2), suggesting that E protein-based assays are more sensitive than NS1-based assays for DENV and WNV. The sensitivity of our ZIKV mutant VLP IgG ELISA (100%) was higher than or compatible to those reported previously (79 to 100%) using the Euroimmun ZIKV NS1 IgG ELISA kit or the blockade of binding ELISA [26–29]. Notably, the specificity of our ZIKV mutant VLP (83.3%) can be further improved to 92.2% by the rOD ratio of mutant to WT VLP (Table 2), which is comparable with that of the ZIKV NS1 blockade of binding ELISA (91.4−92.6%) [29]. Compared with a recent report of IgG ELISA based on mutant recombinant E proteins [37], which had a sensitivity/specificity, after pre-incubation with heterologous antigen, of 100/97.1% and 100/97.9% for ZIKV and DENV, respectively, our IgG ELISA using mutant VLP and rOD ratio had comparable sensitivity and specificity. In addition, our IgM ELISA using mutant VLP had a sensitivity and specificity higher than or comparable to those reported previously using mutant VLP or recombinant E proteins [34,37].

It is worth noting that our VLP-based ELISA can discriminate different ZIKV, DENV and WNV infections, whereas PRNT can only confirm pZIKV infection rather than those experiencing previous flavivirus infections [3]. A recent study suggested neutralizing antibody titres can distinguish ZIKV and DENV infections [56], nonetheless, the sensitivity and specificity of neutralizing antibody titres to discriminate various DENV and ZIKV infections, especially between sDENV and ZIKVwprDENV panels, remains to be determined. Neutralization tests are time-consuming and can be performed only in reference laboratories [3]. Compared with PRNT, our combined WT and mutant VLP IgG ELISA require less time (7 h vs. 5–6 days for PRNT) and less sample volume (6 µL vs. 128 µL for PRNT for 6 antigens or viruses). The VLP-based serological tests could be applied to serodiagnosis, blood screening and serosurveillance for three flavivirus infections, as well as retrospective study of ZIKV infection among pregnant women with CZS. They can also be developed into high-throughput formats such as MIA or rapid tests. These together would enhance our understanding of the epidemiology, pathogenesis and complications of ZIKV in flavivirus endemic regions [1,2].

This study has several limitations. First, in order to discriminate ZIKV and flaviviruses of other serocomplexes such as DENV, we only employed DENV1 VLP and pDENV1 panel to represent DENV antigen and primary DENV infection samples, respectively. Although distinguishing DENV infections by different serotypes is beyond the scope of the study, future studies to include primary DENV infection panels from other serotypes are needed to validate these observations. Second, the sample size in each panel with RT-PCR-confirmed flavivirus infection is small and follow-up samples are limited. Despite two-time point samples for the pZIKV and ZIKVwprDENV panels were available, future studies involving larger sample size and more sequential samples are needed. Third, convalescent-phase samples from sDENV panel were not included the IgG ELISA due to insufficient volume. Whether our FL-mutant VLP can reduce the cross-reactivity of early convalescent-phase samples from sDENV infection remains to be investigated. Fourth, despite an algorithm based on our test to distinguish three flavivirus infections was proposed, more assays that can discriminate other medically important flaviviruses including JEV, YFV and tick-borne encephalitis virus (TBEV) remains to be investigated [57,58]. Additionally, including samples with well-documented repeated flavivirus infections are important for the assay development. As several flavivirus vaccines and vaccine trials have been employed in endemic regions, serological tests that can discriminate ZIKV infection and DENV, JEV, YFV or TBEV vaccinations remain to be exploited [57,58].

## Supplementary Material

Supplemental Material
