# Supplementary material for: Enzyme-linked immunosorbent assays using virus-like particles containing mutations of conserved residues on envelope protein can distinguish three flavivirus infections
Source: Emerg Microbes Infect. 2020 Jul 28;9(1):1722–32. doi: 10.1080/22221751.2020.1797540 (PMC7473235; doi:10.1080/22221751.2020.1797540)
Supplement: Supplemental Material [file TEMI_A_1797540_SM3102.pdf]

Table S1. Sampling time, serotypes and sources of different serum/plasma panels

Panel[a]	No. of subjects /samples	Category (No. of subjects/samples)	Sampling time PSO mean and [range]	Sources (No.) of samples and year
pDENV1	21/25[b]	convalescent (12/13)	47 [19−89] days	Taiwan (6), 2006-9
		post-convalescent (9/12)	6.8 [3−15] months	Hawaii (9), 2015<br>Nicaragua (6), 2006-8
pWNV	36/36	early convalescent (36/36)	not applicable[c]	U.S. ARC, 2006-15
pZIKV	20/38[b]	convalescent (20/20)	17 [14−24] days	Nicaragua, 2016
		post-convalescent (18/18)	6.9 [6−8] months	
ZIKVwprDENV	20/35[b]	convalescent (20/20)	16 [14−19] days	Nicaragua, 2016
		post-convalescent (15/15)	7.0 [6−8] months	
sDENV	44/44	convalescent (24/24)	14 [8−35] days	Taiwan, 2001-2
		post-convalescent (20/20)	9.4 [3−12] months	Taiwan (2), 2006-9<br>Nicaragua (18), 2006-8
flavivirus-naïve	66/66	seroprevalence study	not applicable	Taiwan (66), 2015-16

[a]pDENV1: primary dengue virus type 1 infection; PSO: post-symptom onset; pWNV: primary West Nile virus infection; pZIKV: primary Zika virus infection; sDENV: secondary dengue virus infection; ZIKVwprDENV: Zika virus infection with previous dengue virus infection.
[b]20 subjects from each panel provided convalescent-phase samples and some of them also provided post-convalescent-phase samples.
[c]Index samples tested positive for WNV transcription-mediated amplification, IgM and IgG from blood donors at the American Red Cross (ARC).

Table S2. Plasmid constructs generated in this study

Plasmids[a]	description	VLP generated
pCB-D1	Expresses DENV1 (Hawaii) prM/E proteins.	DENV1 WT-VLP
pCB-D1-FL	Expresses DENV1 (Hawaii) prM/E proteins with FL mutations (W101A+F108A).	DENV1 FL-VLP
pCB-WNV	Expresses WNV (NY99) prM/E proteins.	WNV WT-VLP
pCB-WNV-FL	Expresses WNV (NY99) prM/E proteins with FL mutations (W101A+F108A).	WNV FL-VLP
pCB-ZIKV	Expresses ZIKV (PRVABC59) prM/E proteins.	ZIKV WT-VLP
pCB-ZIKV-FL/BCL	Expresses ZIKV (PRVABC59) prM/E proteins with FL/BCL mutations (W101A+F108A and T76A+Q77A+G78A).	ZIKV FL/BCL-VLP
pCB-ZIKV-FL	Expresses ZIKV (PRVABC59) prM/E proteins with FL mutations (W101A+F108A).	ZIKV FL-VLP
pCB-ZIKV-FL/4BCL	Expresses ZIKV (PRVABC59) prM/E proteins with FL/4BCL mutations (W101A+F108A and T76A+Q77A+G78A+E79A).	ZIKV FL/4BCL-VLP
pCB-ZIKV (MR766)	Expresses ZIKV (MR766) prM/E proteins.	ZIKV (MR766)-VLP

[a]BCL: BC loop; DENV1: dengue virus type 1; E: envelope; FL: fusion loop; prM: premembrane; VLP: virus-like particles; WNV: West Nile virus; WT: wild type; ZIKV: Zika virus.

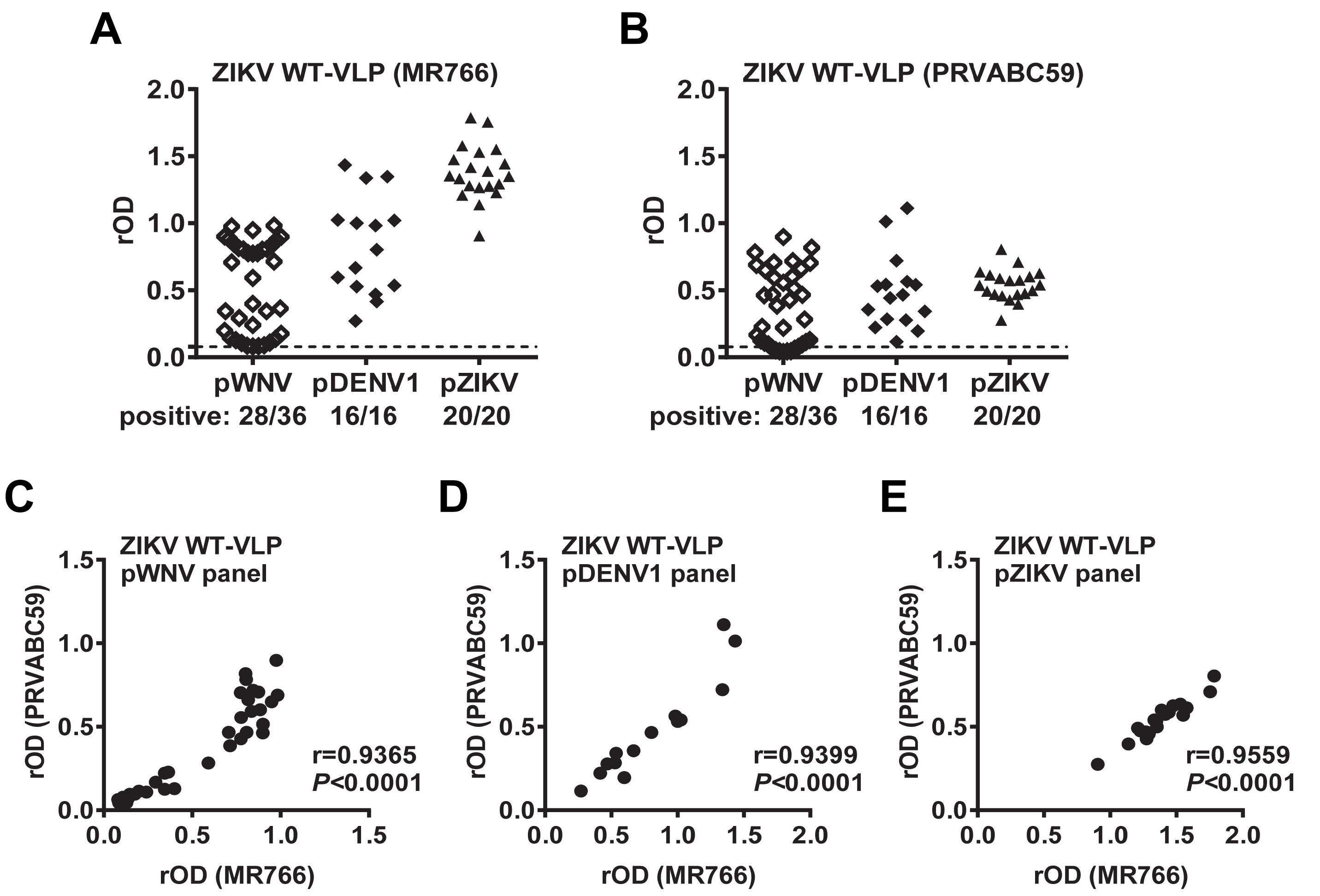

Figure S1. IgG ELISA based on VLP of two ZIKV strains. Convalescent-phase serum or plasma samples from different panels were tested with WT-VLP of ZIKV MR766 strain (A) and PRVABC59 strain (B). Correlation between rOD based on MR766 strain and that on PRVABC59 strain WT-VLP for pWNV (C), pDENV1 (D) and pZIKV (E) panels. Data are the means of two experiments (each in duplicate). Dashed lines indicate cutoff rOD.

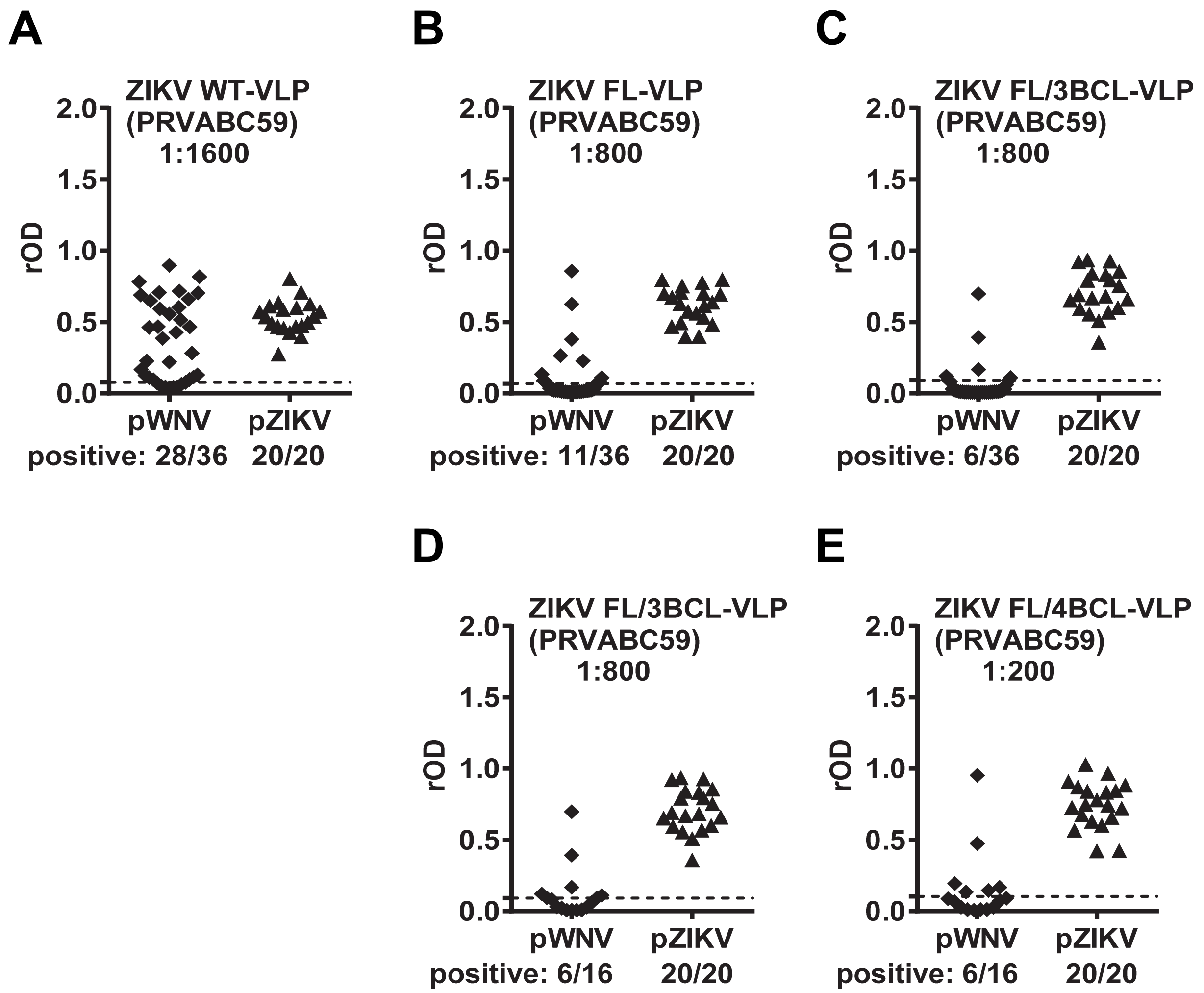

Figure S2. IgG ELISA based on WT, FL and FL/BCL mutant VLP of ZIKV PRVABC59 strain. Convalescent-phase serum or plasma samples from different panels were tested with ZIKV PRVABC59 strain WT (A), FL (B), FL/3BCL (C,D) and FL/4BCL (E) VLP. Data are the means of two experiments (each in duplicate). Dashed lines indicate cutoff rOD.

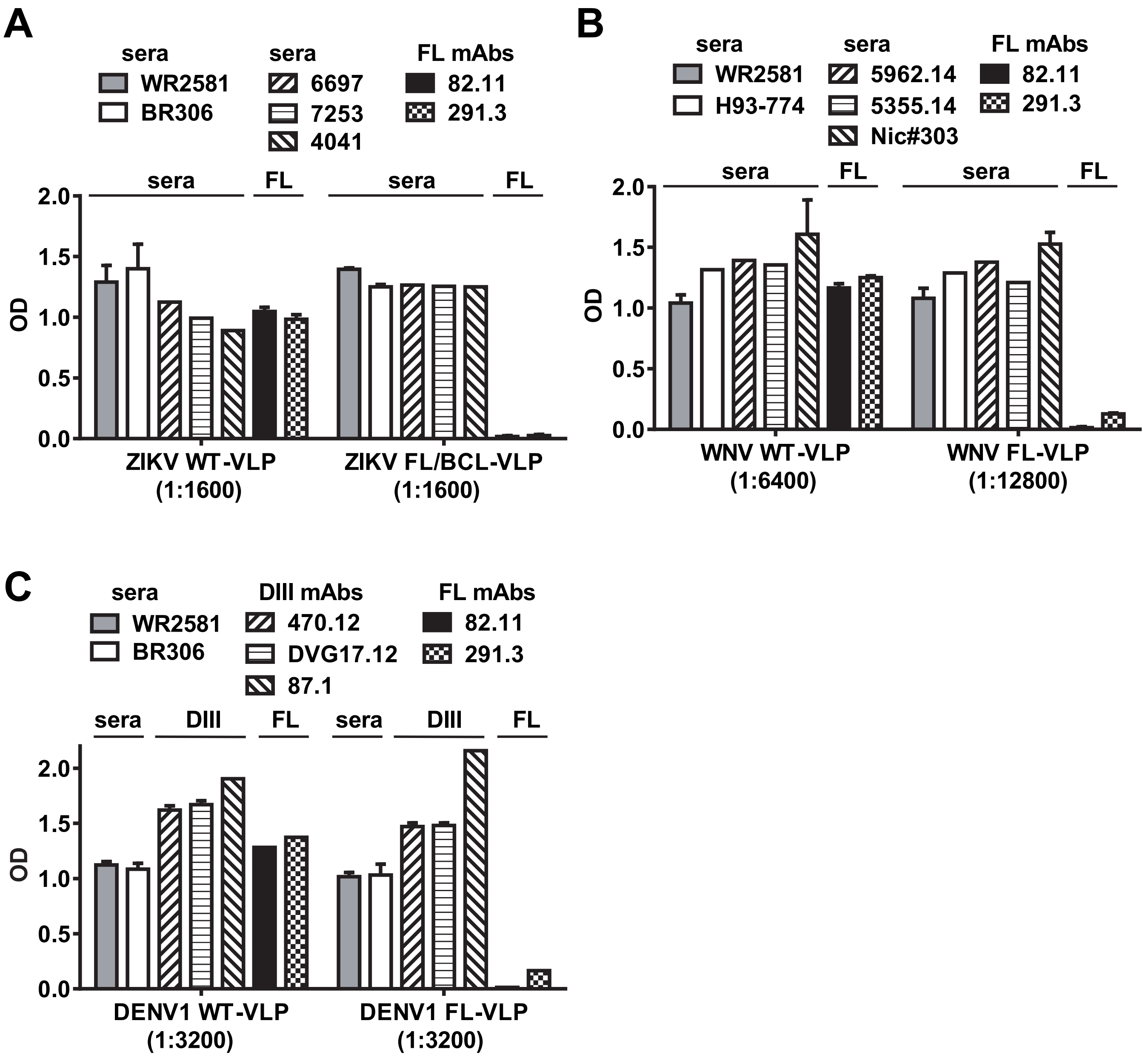

Figure S3. Comparison between the binding of WT- and mutant-VLP by flavivirus-immune sera and mAbs using IgG ELISA. (A) ZIKV WT-VLP and FL/BCL-VLP. (B) WNV WT-VLP and FL-VLP. (C) DENV1 WT-VLP and FL-VLP. The dilutions of WT- and mutant-VLP are shown. Data are the means of two experiments (each in duplicate). Flavivirus-immune sera are from Table S1. Human DIII and FL mAbs have been previously described [51].

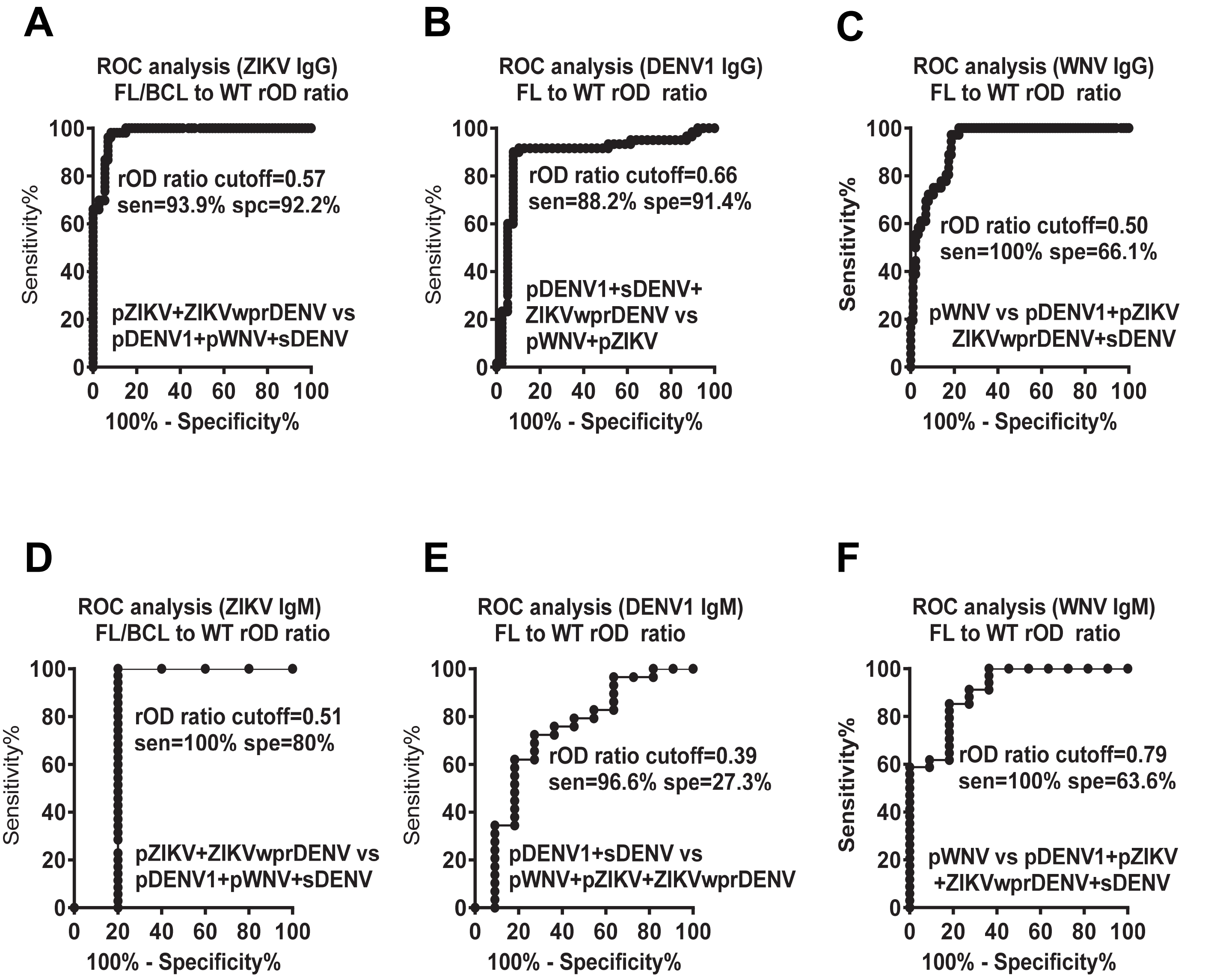

Figure S4. Determination of the cutoff values of the rOD ratio of mutant to WT- VLP by ROC analysis. (A) ZIKV, (B) DENV1, and (C) WNV IgG ELISAs. (D) ZIKV, (E) DENV1, and (F) WNV IgM ELISAs.
